# *In Silico* Comparison of Bioactive Compounds Characterized from *Azadirachta indica* with an FDA-Approved Drug against Schistosomal Agents: New Insight into Schistosomiasis Treatment

**DOI:** 10.3390/molecules29091909

**Published:** 2024-04-23

**Authors:** Babatunji Emmanuel Oyinloye, David Ezekiel Shamaki, Emmanuel Ayodeji Agbebi, Sunday Amos Onikanni, Chukwudi Sunday Ubah, Raphael Taiwo Aruleba, Tran Nhat Phong Dao, Olutunmise Victoria Owolabi, Olajumoke Tolulope Idowu, Makhosazana Siduduzile Mathenjwa-Goqo, Deborah Tolulope Esan, Basiru Olaitan Ajiboye, Olaposi Idowu Omotuyi

**Affiliations:** 1 Phytomedicine, Biochemical Toxicology and Biotechnology Research Laboratories, Department of Biochemistry, College of Sciences, Afe Babalola University, PMB 5454, Ado-Ekiti 360001, Nigeria; 2 Biotechnology and Structural Biology (BSB) Group, Department of Biochemistry and Microbiology, University of Zululand, KwaDlangezwa 3886, South Africa; 3 Institute of Drug Research and Development, S.E. Bogoro Center, Afe Babalola University, PMB 5454, Ado-Ekiti 360001, Nigeria; 4 Department of Pharmacognosy and Natural Products, College of Pharmacy, Afe Babalola University, Ado-Ekiti 360001, Nigeria; 5 College of Medicine, Graduate Institute of Biomedical Sciences, China Medical University, Taichung 40402, Taiwan; 6 Department of Epidemiology and Biostatistics, College of Public Health, Temple University, Philadelphia, PA 19121, USA; 7 Department of Physiology, East Carolina University, Greenville, NC 27834, USA; 8 Graduate Institute of Integrated Medicine, College of Chinese Medicine, China Medical University, Taichung 40402, Taiwan; 9 Department of Traditional Medicine, Can Tho University of Medicine and Pharmacy, Can Tho 900000, Vietnam; 10 Medical Biochemistry Unit, College of Medicine and Health Sciences, Afe Babalola University, PMB 5454, Ado-Ekiti 360001, Nigeria; 11 Industrial Chemistry Unit, Department of Chemical Sciences, College of Sciences, Afe Babalola University, Ado-Ekiti 360001, Nigeria; 12 Faculty of Nursing Sciences, College of Health Sciences, Bowen University, Iwo 232102, Nigeria; 13 Phytomedicine and Molecular Toxicology Research Laboratory, Department of Biochemistry, Federal University Oye-Ekiti, Oye-Ekiti 371104, Nigeria; 14 Department of Pharmacology and Toxicology, College of Pharmacy, Afe Babalola University, PMB 5454, Ado-Ekiti 360001, Nigeria

**Keywords:** dihydroorotate dehydrogenase, *Azadirachta indica*, schistosomiasis, simulation, pharmacophore

## Abstract

The burden of human schistosomiasis, a known but neglected tropical disease in Sub-Saharan Africa, has been worrisome in recent years. It is becoming increasingly difficult to tackle schistosomiasis with praziquantel, a drug known to be effective against all *Schistosoma species*, due to reports of reduced efficacy and resistance. Therefore, this study seeks to investigate the antischistosomal potential of phytochemicals from *Azadirachta indica* against proteins that have been implicated as druggable targets for the treatment of schistosomiasis using computational techniques. In this study, sixty-three (63) previously isolated and characterized phytochemicals from *A. indica* were identified from the literature and retrieved from the PubChem database. In silico screening was conducted to assess the inhibitory potential of these phytochemicals against three receptors (*Schistosoma mansoni* Thioredoxin glutathione reductase, dihydroorotate dehydrogenase, and Arginase) that may serve as therapeutic targets for schistosomiasis treatment. Molecular docking, ADMET prediction, ligand interaction, MMGBSA, and molecular dynamics simulation of the hit compounds were conducted using the Schrodinger molecular drug discovery suite. The results show that Andrographolide possesses a satisfactory pharmacokinetic profile, does not violate the Lipinski rule of five, binds with favourable affinity with the receptors, and interacts with key amino acids at the active site. Importantly, its interaction with dihydroorotate dehydrogenase, an enzyme responsible for the catalysis of the de novo pyrimidine nucleotide biosynthetic pathway rate-limiting step, shows a glide score and MMGBSA of −10.19 and −45.75 Kcal/mol, respectively. In addition, the MD simulation shows its stability at the active site of the receptor. Overall, this study revealed that Andrographolide from *Azadirachta indica* could serve as a potential lead compound for the development of an anti-schistosomal drug.

## 1. Introduction

Human schistosomiasis, commonly known as bilharzia, is a major cause of morbidity in sub-Saharan Africa. This disease is triggered by blood-dwelling parasitic flatworms of the genus Schistosoma. The complex life cycles of Schistosoma species include the infection of both a mammalian definitive host, such as humans, and a freshwater snail as an intermediate host. Restricted availability and access to healthcare facilities, growing poverty, poor sanitary infrastructure, and contact with water activities, especially swimming and irrigated farming, among other factors, predispose people to this debilitating disease [1,2]. In terms of the economic impact of parasite infections that warrant considerable concern, schistosomiasis is the second most significant human parasitic disease after malaria. It accounts for over 280,000 deaths each year, while approximately 250 million people are infected with schistosomiasis annually [3,4].

Research from the World Health Organization (WHO) 2013 indicates that more than 40% of cases of schistosomiasis and other terrestrial parasite diseases, excluding malaria, are caused by skin-penetrating cercariae that are present in contaminated water [5,6].

Additionally, studies reveal that schistosomiasis affected over 229 million people in 2015, thereby accounting for a high percentage of those cases in sub-Saharan Africa between the ages of 5 and 14 years [7,8]. Ameliorating the schistosomiasis challenge was a target for 2020 for the WHO, with the main target, point of action, and milestones focused on scaling up the work to overcome the global effect posed by schistosomiasis. Moreover, with few successes recorded in eradicating schistosomiasis in most countries such as Japan, Tunisia, and some Caribbean countries, more countries like China, Egypt, and Nigeria are taking steps to eradicate the disease as well [9]. Among the vulnerable to schistosomiasis are poor people from rural areas, especially in regions where farming and fishing are the main industries. Household practices like clothes washing in this area also predispose one to contact with contaminated water and risk of infection [10]. Children are also more susceptible to schistosomiasis because of poor hygiene and recreational activities like swimming [11,12].

Common species of schistosomes are *S. mansoni*, *S. haematobium*, *S. japonicum*, *S. mekongi*, and *S. intercalatum*. Intestinal schistosomiasis is typically more prevalent in sub-Saharan Africa due to the presence of *S. mansoni* and *S. haematobium*, among other parasites [7]. *S. mansoni*, a cause of liver and intestine schistosomiasis in South America and Africa, is spread by biomphalaria snails. While biomphalaria snails include many species, such as *B. alexandrina*, *B. sudanica*, *B. pfeifferi*, and *B. hoanomphala*, the genus Bulinus contains the following species: *B. tropicus*, *B. globosus*, *B. truncatus*, *B. forskalli*, and *B. africanus*. *S. haematobium* is a major cause of urinary schistosomiasis [11,13,14]. The microscopic examination of faeces and urine remains the most reliable method for the diagnosis of schistosomal infection. The distinctive size, shape, and presence of lateral or terminal spines make schistosome eggs easily identifiable when observed under a microscope [15,16].

Praziquantel has been one of the treatment choices for schistosomiasis since the mid-1980s. However, compared to praziquantel, which targets adult parasites, the new schistosomiasis medication Artemether targets larvae parasites more efficiently. As a result, Artemether’s use for treating schistosomiasis should be limited to prevent the development of drug resistance in the malaria parasite [17,18,19]. Major concerns are raised by frequent reports of resistance to oxamniquine, another authorized drug that is used to treat *S. mansoni* [20,21]. The difficulty of dealing with the parasite and the pharmaceutical industry’s habitual indifference to tropical diseases prevent the development of a novel strategy for antischistosomal medication research.

Several chemical groups, including oxadiazole 2-oxides, phosphinic acid amides, isoxazolones, and phosphoramidites, have been investigated by scientists, and it has been demonstrated that they suppress smTGR activity. It was discovered that 4-Phenyl-1,2,5-oxadiazole-3-carbonitrile-2-oxide is active against TGR from smTGR and that it inhibits thioredoxin glutathione reductase (TGR) from *S. japonicum* in the low nanomolar range [22].

Due to its therapeutic and pesticidal properties, the traditional medicinal plant *Azadirachta indica* possesses the therapeutic potency to treat several human illnesses and act against organisms that are drug-resistant and capable of forming biofilms, such as Schistosomiasis. It also has pharmacological significance and is a highly rich source of bioactive ligands. *A. indica* has been studied in both experimental and clinical settings with an active chemical isolated from the *A. indica* tree and has been recognized for some years to have antiviral, antifungal, antibacterial, and insecticidal properties among natural products [22,23,24]. The plant has also traditionally been used for parasite infection treatment and gastrointestinal problems [25,26]; however, an effort to drastically control schistosome infections is still based largely on the administration of a chemotherapeutic agent such as praziquantel (PZQ), an oral therapy that is both safe and effective against all human schistosome species. Unfortunately, fast re-infection following treatment with PZQ, as well as certain cases of PZQ resistance in the treatment of specific strains of *S. haematobium* and *S. mansoni* as a result of drug pressure, remain a concern. Due to the aforementioned, it has become vital to look for a new antischistosomal drug to replace praziquantel therapy. In this study, we validated the anti-schistosomal activity of compounds identified from *A. indica* using various in silico tools.

## 2. Results and Discussion

Bioinformatics has provided a rapid and versatile platform for drug discovery and the investigation of diseases at the molecular level via the use of computational techniques. This platform has played a key role in the understanding of protein–ligand interaction, which is critical for translational drug development [27,28,29,30,31]. There has recently been an upsurge of interest in the exploration of small molecules as an alternative therapy for schistosomiasis treatment, with current therapies showing inadequacy in the treatment and ameliorating the resurgence and spread of this disease [32,33]. Therefore, the search for a novel antischistosomal drug to use as an alternative to praziquantel therapy has become necessary.

This work, therefore, explored the therapeutic potential of compounds identified from *Azadirachta indica* as novel anti-schistosomal lead compounds using a computational screening approach [34,35,36]. Three clinical targets, namely *Schistosoma mansoni* Thioredoxin glutathione reductase, dihydroorotate dehydrogenase, and Arginase, were chosen based on their key roles in the maintenance of redox homeostasis, the pyrimidine nucleotide biosynthetic pathway, and metabolism, respectively. The intermolecular interaction of the top three hit compounds (5318517: Andrographolide; 5318767: Nicotiflorin; and 5280804: Isoquercitrin) as well as standard drugs 4891: Praziquantel (a conventional schistosomal medication), 4612: Oxamniquine, and 68827: Artemisinin were studied.

**Figure 1:**
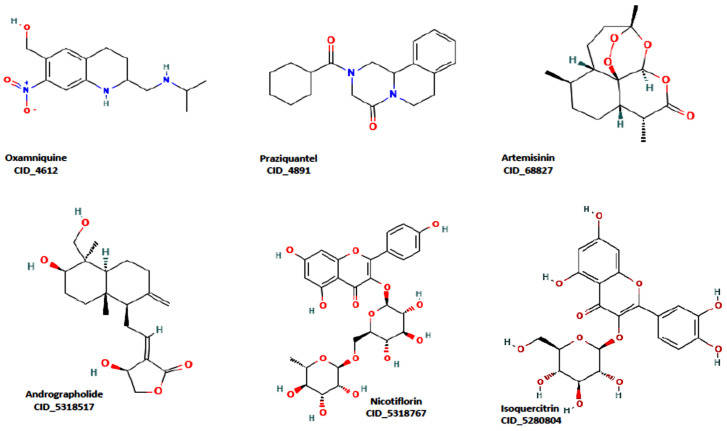
The three reference ligands, Oxamniquine, Praziquantel, and Artemisinin, and identified ligands (Andrographolide, Nicotiflorin, and Isoquercitrin).

Figure 1 shows the structure of the hit compounds and that of the standard drugs on the market. The pharmacokinetic profile and drug-likeness of a compound have been identified as key factors in identifying potential lead compounds. High/good gastrointestinal absorption is desirable in oral formulations and therefore plays an important role in the delivery of the drug to the target site [37,38]. These properties are presented in Table 1, Table 2 and Table 3 for the hit compounds and the standards. The results of the ADMET analysis (Table 1, Table 2 and Table 3) show that Andrographolide has a favourable profile, with high gastrointestinal absorption, and conforms with Lipinski’s rule (zero violation), like the standard drugs, in comparison with other hit compounds (Nicotiflorin and Isoquercitrin have low gastrointestinal absorption profile), as shown in Table 3. In general, the drug-likeness and ADMET profile of Andrographolide and the standard drug, Pranziquantel, are similar. Moreover, Andrographolide is not a BBB permeant, and does not inhibit CYP enzymes. This makes Andrographolide a promising compound for further analysis.

The docking score could be used to predict the inhibitory potentials of ligands at the receptor site [39,40,41]. Thus, to predict the therapeutic efficacy of the hit compounds, molecular docking with the selected proteins was carried out using the Schrodinger’s Maestro suite (Glide), and the observed interactions are shown in Table 4. As presented in Table 4, these results suggest a good binding affinity of the hit compounds to the active site of the target proteins. Andrographolide emerged as the best candidate, not just because of its superior glide score, but because of its favourable pharmacokinetic profiles, as discussed and shown in Table 1, Table 2 and Table 3. The intermolecular interaction of this compound was compared to Pranziquantel. The SmTGR-Andrographolide complex showed a glide score of −4.65 kcal/mol and MMGBSA of −30.49 kcal/mol, with the formation of three hydrogen bonds with SER85, ASP84, and GLN86 residues. However, Praziquantel has a glide score of −2.58 kcal/mol, MMGBSA of −28.18 kcal/mol, and one hydrogen bond with SER85 residue.

In addition, the SmDHODG–Andrographolide complex had −10.19 kcal/mol docking energy and −45.75 kcal/mol free binding energy. It was also able to produce a similar pose to the co-crystalized inhibitor and interact with similar amino acid residues at the active site, forming hydrogen bonds with PHE61, ARG130, ALA49, and SER53. These, in addition to its hydrophobic interactions, were projected to provide complex stability when compared to Praziquantel. *Schistosoma mansoni’s* arginase–andrographolide complex, on the other hand, had a glide score of −4.34 kcal/mol and hydrogen bonds with ASP211, SER165, and ASN160 when compared with Praziquantel complex with a glide score of −3.41 kcal/mol and a hydrogen bond with SER167, as shown in Table 4. The docking scores observed for Andrographolide across these receptors are comparable with known inhibitors from the CHEMBL database (Table 5 and Table 6).

The docking validation and ligand interactions for these complexes are shown in Figure 2, Figure 3, Figure 4, Figure 5, Figure 6, Figure 7, Figure 8, Figure 9, Figure 10, Figure 11 and Figure 12.

**Figure 13:**
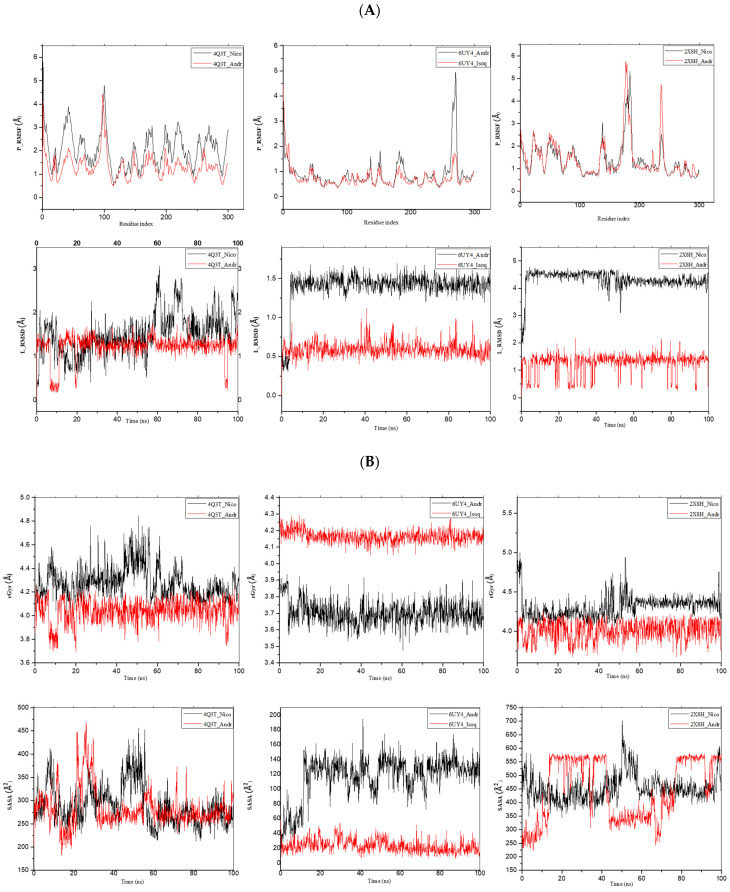
MD simulation of 4Q3T, 6UY4, and 2X8H complexed to 5318517: Andrographolide, 5318767: Nicotiflorin, and 5280804: Isoquercitrin. (**A**) RMSF and RMSD graphical illustration plot. (**B**) rGyr representation and SASA diagram. All simulations were carried out using Schrödinger’s Maestro suite.

Following a promising result from the molecular docking, Molecular Dynamic (MD) Simulation analysis was performed for 100 ns to evaluate the atomistic level changes in receptors’ binding sites and the stability of the ligands at the binding site of the receptor with respect to the time scale. The root mean square deviation (RMSD), root mean square fluctuation (RMSF), radius of gyration (rGyr), pressure swing adsorption (PSA), and solvent-accessibility surface area (SASA) were analysed, as shown in Table 5 (mean ± SEM) and Figure 13.

The RMSD data give valuable information on variability when applied to very similar proteins. They reveal the conformational variation of a protein–ligand complex over time. The lower the value, the better the stability of the protein–ligand complex. The MD simulation data, as shown in Figure 13, reveal Andrographolide’s stable binding conformation with the receptors, with average RMSD values of 1.151, 1.301, and 1.137, respectively, as shown through the 100 ns MD simulation. The RMSF indicates the crucial residues involved in the strongest interactions with a ligand, while the rGyr was analysed to deduce their changes in compactness and to ascertain the protein’s stability. Figure 13B reveals the gyration radius spectrum of the Andrographolide complexes with 4Q3T, 6UY4, and 2X8H, with an average rGyr of 4.022, 3.728, and 3.399, respectively (Table 7). It is important to note that the Andrographolide complexes are the most compact of all the ligands, having shown the lowest average rGyr value. The plot showed stability throughout the simulation period for Andrographolide-6UY4 and Andrographolide-2X8H, and therefore it could be argued that it does not cause any distortion in the structure of the proteins. This supports our RMSD and molecular docking findings that Andrographolide may be an effective inhibitor of the *Schistosoma mansoni* thioredoxin glutathione reductase and dihydroorotate dehydrogenase.

## 3. Research Methodology

### 3.1. Retrieval of the Ligand and Receptor

A library of sixty-three (63) bioactive compounds (phytochemical) previously characterized from *A. indica* that had been reported in the literature was created. These phytochemicals (ligands) were retrieved from the PubChem database (https://pubchem.ncbi.nlm.nih.gov/ accessed on 25 October 2023). Similarly, the three-dimensional (3D) structure of the selected proteins (receptor)—*S. mansoni* thioredoxin glutathione reductase (PDB ID: 2X8H), *S. mansoni* dihydroorotate dehydrogenase (PDB ID: 6UY4) and *S. mansoni* arginase (PDB ID: 4Q3T)—were downloaded from an online research collaboration for structural bioinformatics (RCSB) protein data bank (https://www.rcsb.org/ accessed on 25 October 2023).

### 3.2. Preparation and Generation of the Protein Grid

The protein preparation and grid generation were carried out using the protein preparation wizard and Glide Grid tools in Maestro’s Schrödinger Suite, as previously described [42,43].

### 3.3. Ligand Preparation

Ligand preparation was conducted by OPLS3 optimization at a physiologically relevant PH range of 7.2 ± 0.2. For each ligand structure, potential ionization states were generated, and stereoisomers were computed by modifying specific chiralities while keeping others constant [44].

### 3.4. Computational Approach in Extra Precision (XP) Docking Analysis and Docking Validation

The library of 63 ligands downloaded from PubChem was used for molecular docking studies to discover potential interactions with the active site of the receptors. First, the 63 compounds were screened using glide high throughput virtual screening (HTVS), and the top 10% of the ranking ligands were chosen for (XP) docking as a scoring algorithm to carry out more upscale docking simulation with the receptors. To validate the molecular docking protocol, the co-crystallised ligands were extracted and redocked to the active site of the receptors [45]. Also, the FASTA sequence of the target proteins was blasted with the CHEMBL database server (https://www.ebi.ac.uk/chembl/ accessed on 3 April 2024). A set of compounds known to have inhibitory activities on these targets was retrieved and docked to the active site to validate the docking protocol.

### 3.5. Absorption, Distribution, Metabolism, Excretion, and Toxicological Prediction

The Qikprop option of the Maestro tools was used to estimate the (ADME) properties and toxicological potency of ligands together with molecular properties of the lead ligands [46].

### 3.6. Free Binding Energy Determination

The Prime MM-GBSA software (https://www.schrodinger.com/platform/) of the Schrödinger suite was used to calculate the potential free binding energy of the receptor–ligand complexes to ascertain the balance of their complexes. The OPLS3 and VSGB were chosen as the force field and solvent model, respectively, with other options set at default settings for the free binding energy calculation. This was calculated using the equation ΔGbind = ΔE + ΔG_solv_ + ΔGSA (1) ΔE = E_complex_ − E_protein_ − E_ligand_, where E_complex_, E_protein_, and E_ligand_ are the minimized energies of the protein–inhibitor complex, protein, and inhibitor, respectively, and ΔG_solv_ = G_solv_(complex) − G_solv_(protein) − G_solv_(ligand), where G_solv_(complex), G_solv_(protein), and G_solv_(ligand) are the solvation free energies of the complex, protein, and inhibitor. ΔGSA = GSA (complex) − GSA (protein) − GSA (ligand), where GSA (complex), GSA (protein), and GSA (ligand) represent the surface area energies for the complex, protein, and inhibitor, respectively [47].

## 4. Conclusions

Based on the molecular docking analysis, this study revealed that some phytochemicals from *Azadirachta indica* possess a similar or even higher affinity for SmTGR, SmDHODG, and *Schistosoma mansoni* Arginase than Praziquantel, the drug available on the market for the treatment of schistosomiasis. The docking analysis shows that Andrographolide, Isoquercitrin, and Nicotiflorin possess a high affinity for these receptors. The MD simulation confirmed this observation by showing that these phytochemicals generated good binding affinity towards the receptors. Andrographolide stands out from all these phytochemicals because of its favourable pharmacokinetic profile and stable binding conformation at the receptor site. Our results therefore suggest that Andrographolide can act as a multi-target inhibitor of *Schistosoma mansoni* key metabolic enzymes. Therefore, this study revealed that Andrographolide from *Azadirachta indica* could serve as a potential lead compound for the development of an anti-schistosomal drug, owing to its satisfactory pharmacokinetic profile, the non-violation of the Lipinski rule of five, its good binding affinity, and stability at the receptor site of the *Schistosoma mansoni* key metabolic enzymes. These make it a viable compound worthy of potential optimization and the experimental evaluation of its biological activity for the possible development of anti-schistosomal drugs. Therefore, in vitro and/or in vivo experimental validation of Andrographolide’s anti-schistosomal activity is recommended.

## Figures and Tables

**Figure 2:**
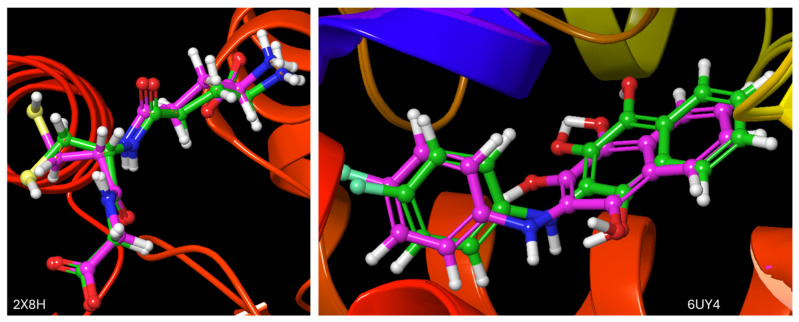
The superimposed structures of the co-crystalised ligands in their co-crystallised (magenta) and re-docked poses (green) [RMSD = 0.73 and 0.87 A for 2X8H and 6UY4, respectively].

**Figure 3:**
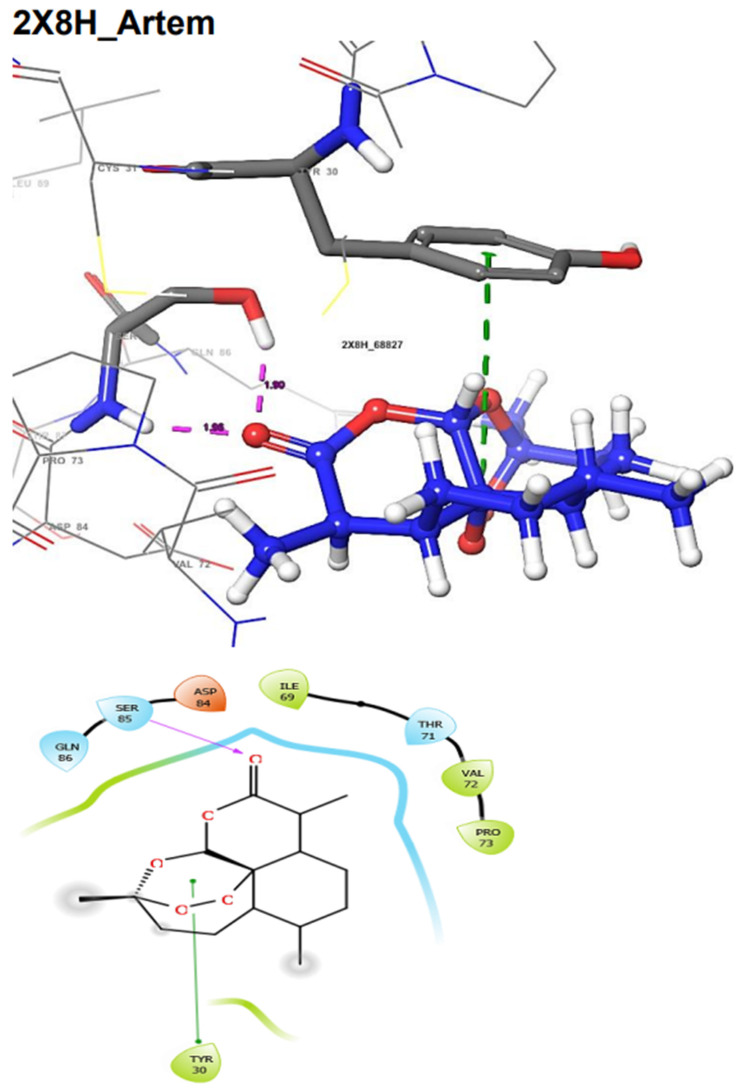
The 3D (**top**) and 2D (**bottom**) ligand interactions of the Artemisinin–thioredoxin glutathione reductase complex. The binding site residues’ charges are indicated in the 2D image by the colours red for negative, blue for positive, and white for neutral.

**Figure 4:**
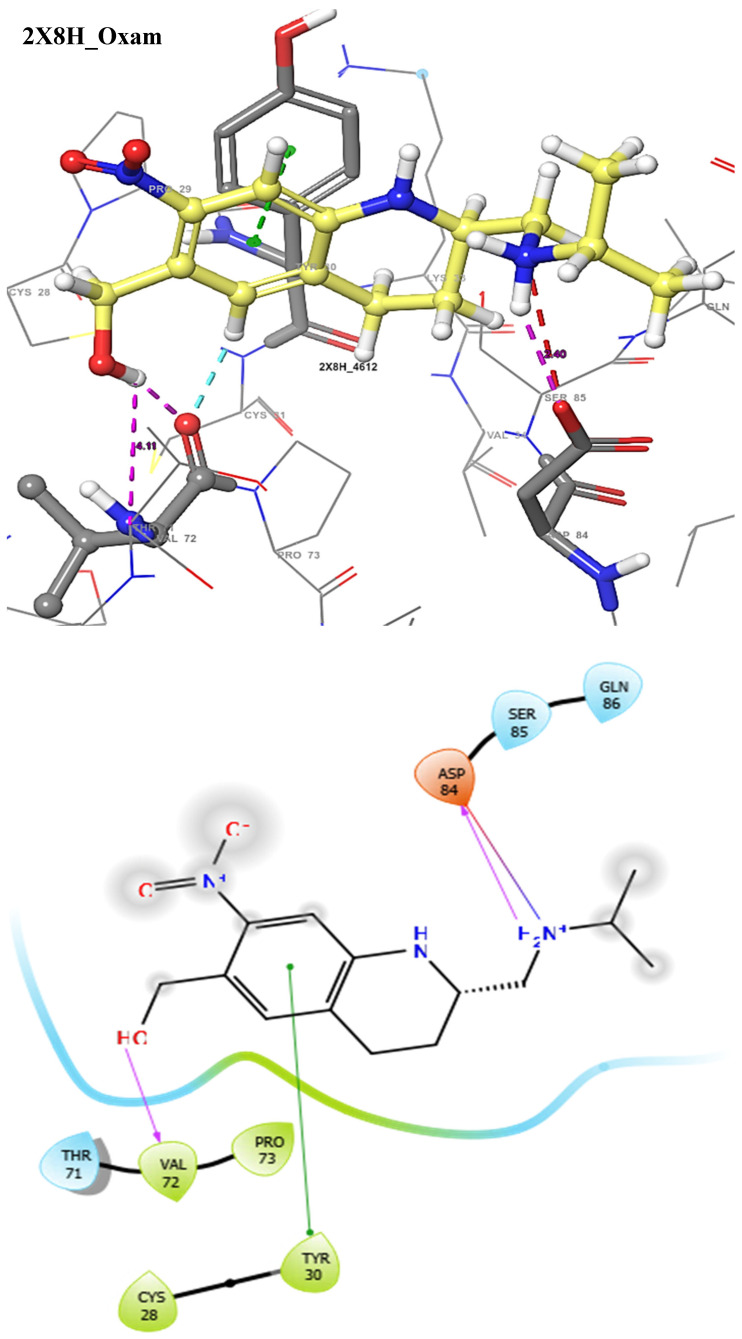
The 3D (**top**) and 2D (**bottom**) ligand interactions of the Oxamniquine–thioredoxin glutathione reductase complex. The binding site residues’ charges are indicated in the 2D image by the colours red for negative, blue for positive, and white for neutral.

**Figure 5:**
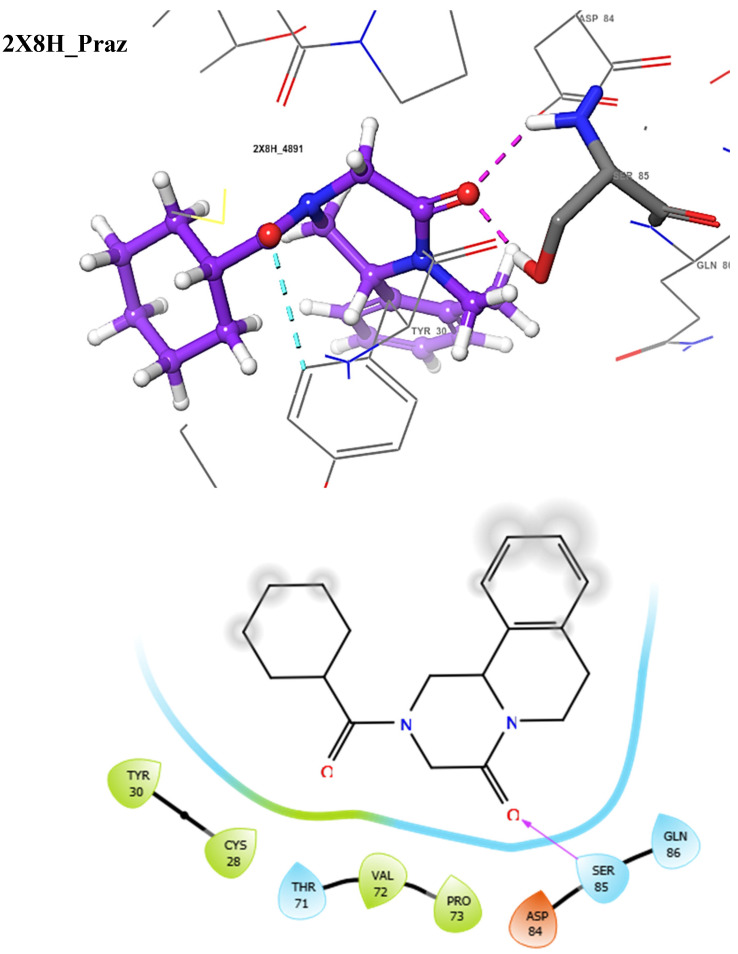
The 3D (**top**) and 2D (**bottom**) ligand interactions of the Praziquantel–thioredoxin glutathione reductase complex. The binding site residues’ charges are indicated in the 2D image by the colours red for negative, blue for positive, and white for neutral.

**Figure 6:**
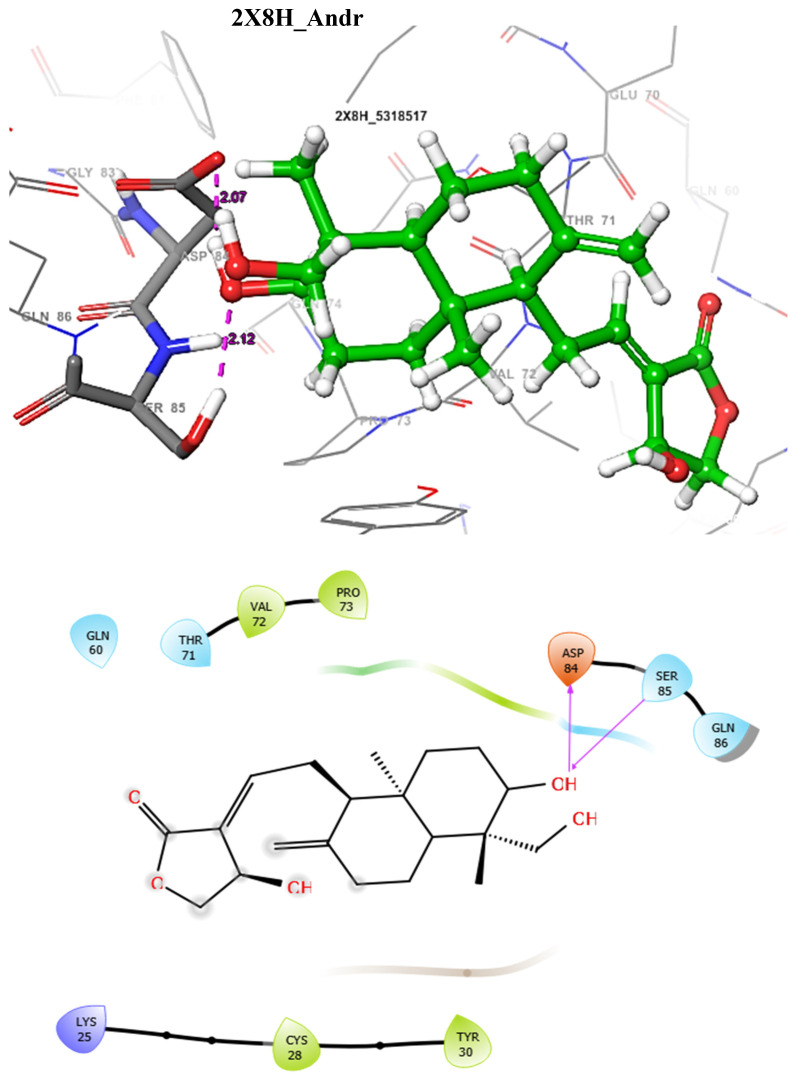
The 3D (**top**) and 2D (**bottom**) ligand interactions of the Andrographolide–thioredoxin glutathione reductase complex. The binding site residues’ charges are indicated in the 2D image by the colours red for negative, blue for positive, and white for neutral.

**Figure 7:**
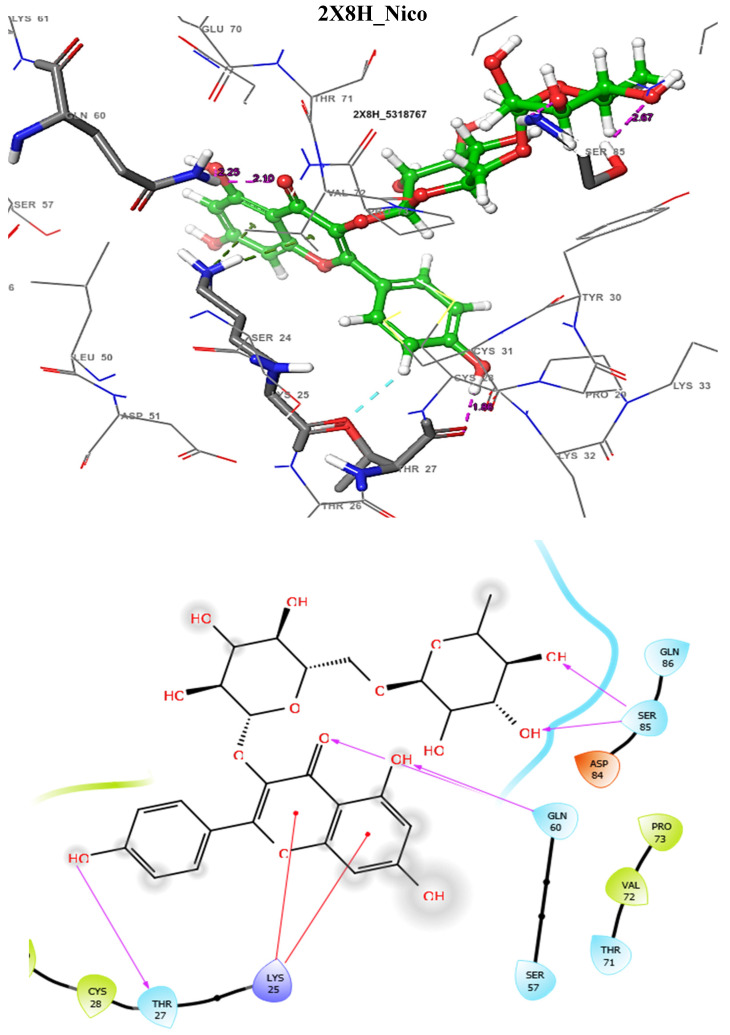
The 3D (**top**) and 2D (**bottom**) ligand interactions of the Nicotiflorin–thioredoxin glutathione reductase complex. The binding site residues’ charges are indicated in the 2D image by the colours red for negative, blue for positive, and white for neutral.

**Figure 8:**
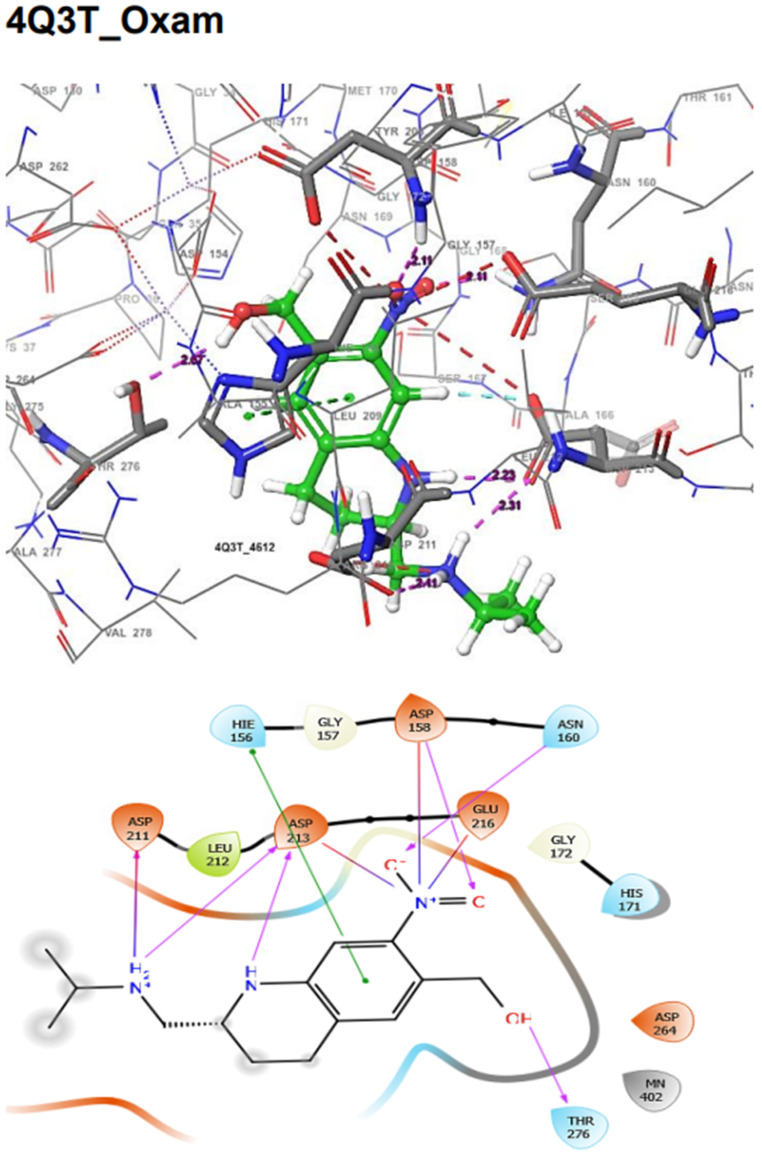
The 3D (**top**) and 2D (**bottom**) ligand interactions of the Oxamniquine–*Schistosoma mansoni* Arginase complex. The binding site residues’ charges are indicated in the 2D image by the colours red for negative, blue for positive, and white for neutral.

**Figure 9:**
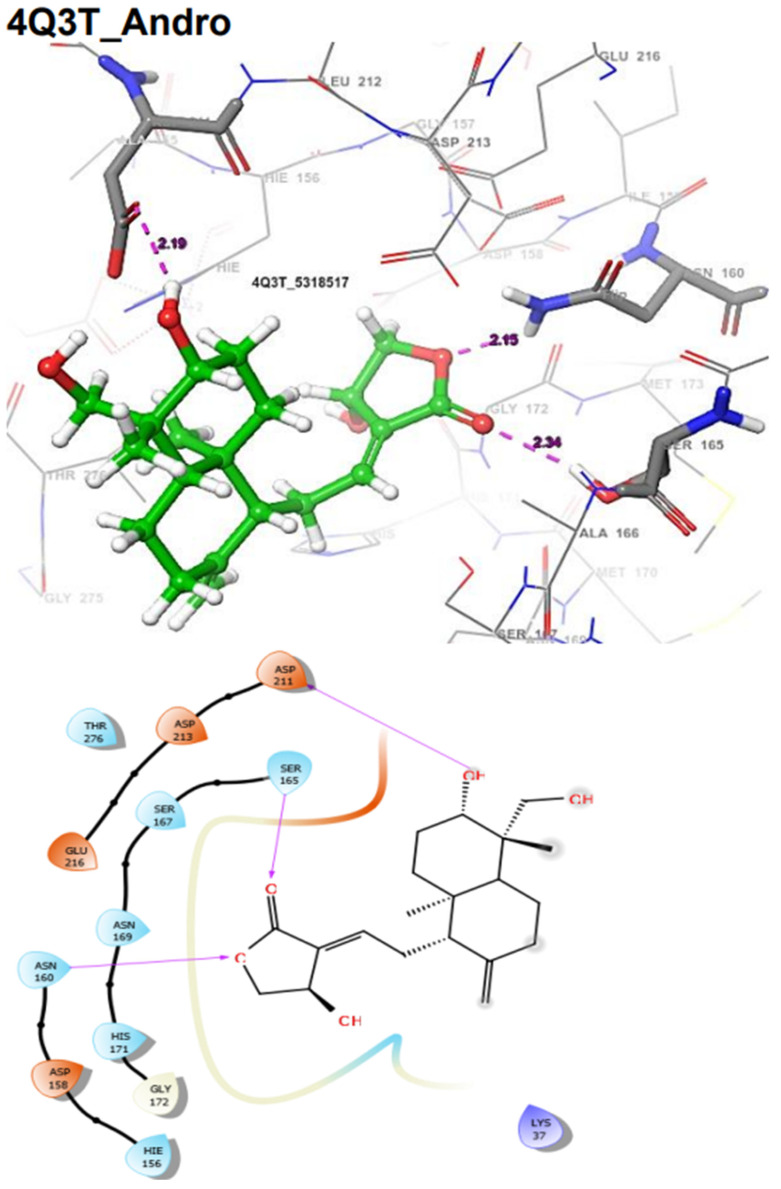
The 3D (**top**) and 2D (**bottom**) ligand interactions of the Andrographolide–*Schistosoma mansoni* Arginase complex. The binding site residues’ charges are indicated in the 2D image by the colours red for negative, blue for positive, and white for neutral.

**Figure 10:**
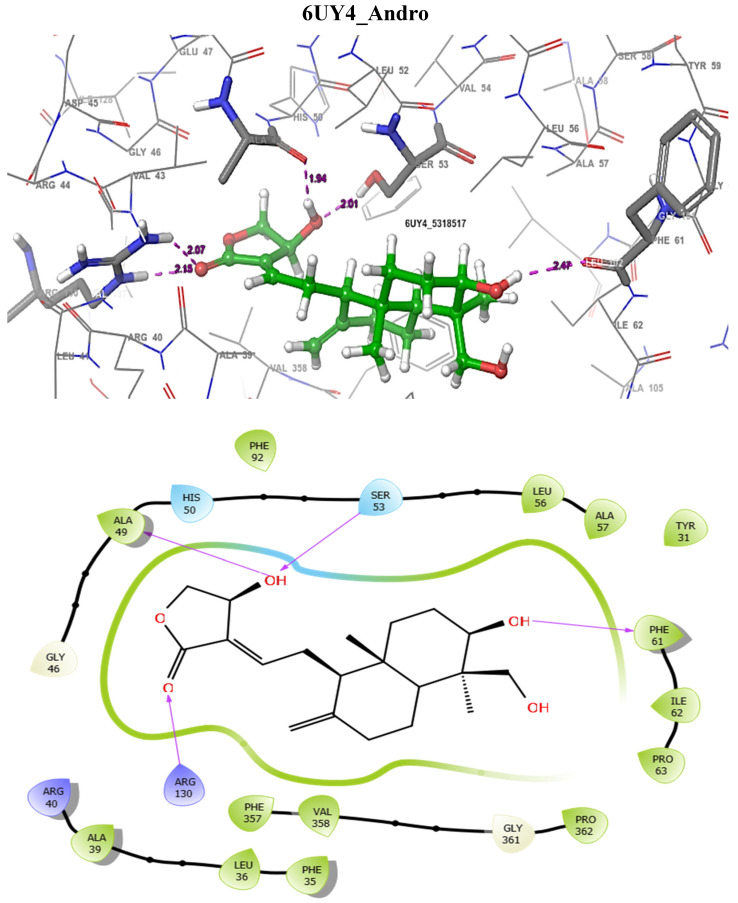
The 3D (**top**) and 2D (**bottom**) ligand interactions of the Andrographolide–*Schistosoma mansoni* dihydroorotate dehydrogenase complex. The binding site residues’ charges are indicated in the 2D image by the colours red for negative, blue for positive, and white for neutral.

**Figure 11:**
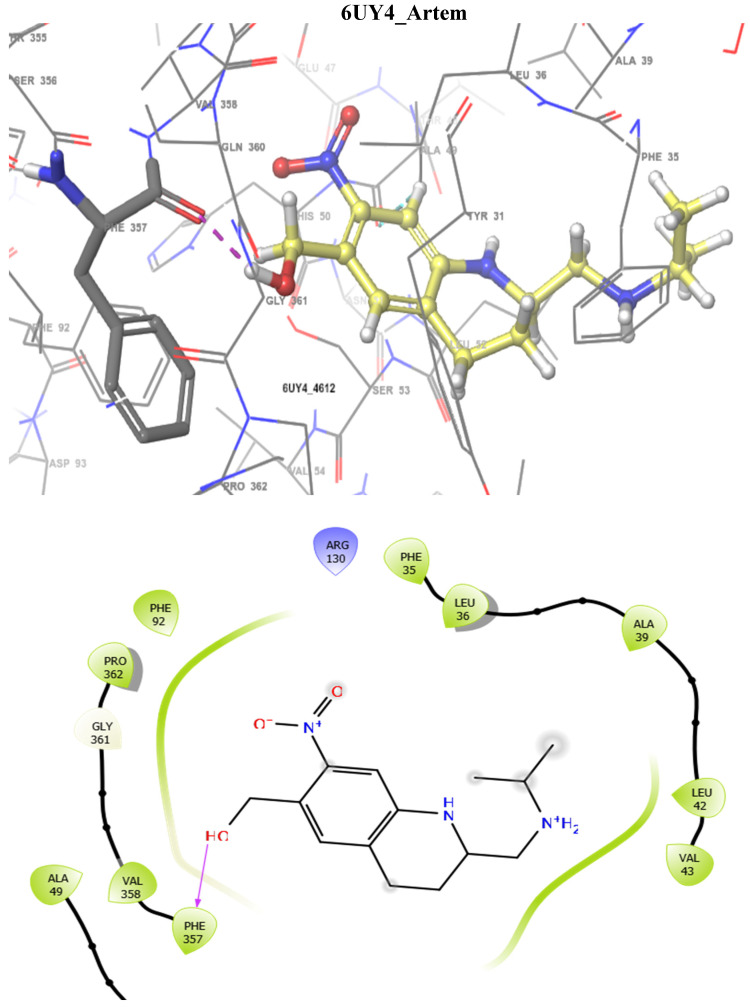
The 3D (**top**) and 2D (**bottom**) ligand interactions of the Artemisinin–*Schistosoma mansoni* dihydroorotate dehydrogenase complex. The binding site residues’ charges are indicated in the 2D image by the colours red for negative, blue for positive, and white for neutral.

**Figure 12:**
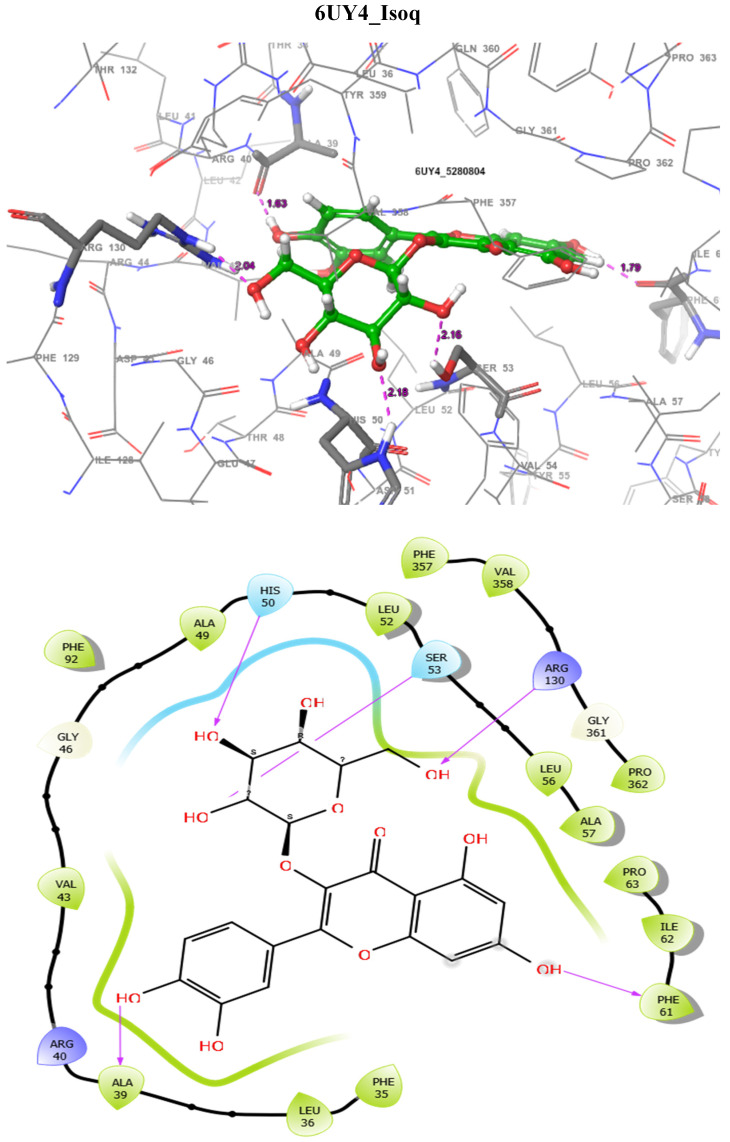
The 3D (**top**) and 2D (**bottom**) ligand interactions of the Isoquercitrin–*Schistosoma mansoni* dihydroorotate dehydrogenase complex. The binding site residues’ charges are indicated in the 2D image by the colours red for negative, blue for positive, and white for neutral.

**Table 1:** Hit compounds from *Azadirachta indica* with their physicochemical properties.

Ligand	MW	#HA	#AHA	#RB	#HBA	#HBD
4612	279.33	20	6	4	5	3
4891	312.41	23	6	2	2	0
68827	282.33	20	0	0	5	0
5318517	350.45	25	0	3	5	3
5318767	594.52	42	16	6	15	9
5280804	464.38	33	16	4	12	8

MW: Molecular weight; #HA: no. of heavy atoms; #AHA: no. of aromatic heavy atoms; #RB: no. of rotatable bonds; #HBA: H-bond acceptors; #HBD: H-bond donors (4612: Oxamniquine; 4891: Praziquantel; 68827: Artemisinin; 5318517: Andrographolide; 5318767: Nicotiflorin; 5280804: Isoquercitrin).

**Table 2:** Medicinal chemistry profile and drug-likeness of reference drugs and hit compounds from *Azadirachta indica*.

Ligand	Lipinski #Violations	Ghose #Violations	Veber #Violations	Egan #Violations	TPSA	BS	PAINS #Alerts
4612	0	0	0	0	90.11	0.55	0
4891	0	0	0	0	40.62	0.55	0
68827	0	0	0	0	53.99	0.55	0
5318517	0	0	0	0	86.99	0.55	0
5318767	3	4	1	1	249.2	0.17	0
5280804	2	1	1	1	210.15	0.17	1

(4612: Oxamniquine; 4891: Praziquantel; 68827: Artemisinin; 5318517: Andrographolide; 5318767: Nicotiflorin; 5280804: Isoquercitrin).

**Table 3:** Absorptivity, distribution, and metabolism properties of identified ligands compared with reference ligands.

Ligand	GI Absorption	BBB Permeant	PGP Substrate	CYP1A2 Inhibitor	CYP2C19 Inhibitor	CYP2C9 Inhibitor	CYP2D6 Inhibitor
4612	High	No	Yes	No	No	No	No
4891	High	Yes	Yes	No	Yes	No	Yes
68827	High	Yes	No	Yes	No	No	No
5318517	High	No	Yes	No	No	No	No
5318767	Low	No	Yes	No	No	No	No
5280804	Low	No	No	No	No	No	No

Note: BBB, blood–brain barrier; GI, gastrointestinal.

**Table 4:** Molecular interaction of the docking output of the complexes.

Receptor	Ligand	Glide Score (kcal/mol)	MMGBSA (kcal/mol)	H-Bond	Salt Bridge	PI-PI	PI Cation
2X8H	68827	−3.86	−33.27	SER85	TYR30	-	-
	4612	−3.49	−31.67	ASP84, VAL72	ASP84	TYR30	-
	4891	−2.58	−28.18	SER85	-	-	-
	5318767	−5.97	−46.40	THR25, GLN60, SER85	-	-	LYS25
	5318517	−4.65	−30.49	SER85, ASP84, GLN86	-	-	-
4Q3T	68827	−2.87	−26.16	-	-	-	-
	4891	−3.41	−33.61	SER167	-	-	-
	4612	−5.97	−28.78	THR276, ASN160, ASP158, ASP213, ASP211	ASP211, ASP213, ASP158	HIS156	-
	5318767	−8.67	−41.44	ASP211, ASP213,ALA156, SER165, GLU216,SER167,	-	-	-
	5318517	−4.34	−30.05	ASP211, SER165, ASN160	-	-	-
6UY4	68827	−5.37	−39.33	-	-	-	-
	4891	−6.80	−40.64	SER53	-	PHE35	-
	4612	−3.23	−44.21	PHE357	-	-	-
	5280804	−12.80	−52.05	HIS50, SER53, ARG130, PHE61, ALA39	-	-	-
	5318517	−10.19	−45.75	PHE61, ARG130, ALA49, SER53	-	-	-

(2X8H: *Schistosoma mansoni* thioredoxin glutathione reductase with GSH in a complex; 6UY4: *Schistosoma mansoni* dihydroorotate dehydrogenase crystal structure; 4Q3T: crystal structure of *Schistosoma mansoni* Arginase 4612: Oxamniquine; 4891: Praziquantel; 68827: Artemisinin; 5318517: Andrographolide; 5318767: Nicotiflorin; 5280804: Isoquercitrin).

**Table 5:** Binding energies of known inhibitors for *Schistosoma mansoni* thioredoxin glutathione reductase (2X8H) retrieved from the CHEMBL database.

S/N	Compounds	Docking Score (Kcal/mol)
1.	CHEMBL3322286	−5.46
2.	CHEMBL1486739	−4.26
3.	CHEMBL3322292	−4.01
4.	CHEMBL1455957	−3.81
5.	CHEMBL4846043	−3.73
6.	CHEMBL1449349	−3.59
7.	CHEMBL568961	−3.46
8.	CHEMBL4858362	−3.41
9.	CHEMBL3322287	−3.36
10.	CHEMBL571936	−3.33
11.	CHEMBL500868	−3.30
12.	CHEMBL567641	−3.27
13.	CHEMBL582970	−3.24
14.	CHEMBL578810	−3.22
15.	CHEMBL1325877	−3.21
16.	CHEMBL576118	−3.14
17.	CHEMBL574577	−3.13
18.	CHEMBL570130	−3.09
19.	CHEMBL1428415	−3.07
20.	CHEMBL4852477	−3.06

**Table 6:** Binding energies of known inhibitors for *Schistosoma mansoni* dihydroorotate dehydrogenase (6UY4) retrieved from the CHEMBL database.

S/N	Compounds	Docking Score (Kcal/mol)
1.	CHEMBL1450	−10.35
2.	CHEMBL4586212	−9.77
3.	CHEMBL38434	−9.51
4.	CHEMBL38434	−9.51
5.	CHEMBL4472078	−9.33
6.	CHEMBL4474026	−9.18
7.	CHEMBL2023282	−9.10
8.	CHEMBL4524841	−9.05
9.	CHEMBL4560384	−8.73
10.	CHEMBL4540838	−8.63
11.	CHEMBL2408379	−8.58
12.	CHEMBL4452960	−8.45
13.	CHEMBL4457147	−8.44
14.	CHEMBL2041119	−8.38
15.	CHEMBL4566973	−8.18
16.	CHEMBL2408378	−8.15
17.	CHEMBL973	−8.13
18.	CHEMBL4546952	−7.99
19.	CHEMBL1738786	−7.94
20.	CHEMBL4527976	−7.81

**Table 7:** Interactive properties of MDs of the native receptors and protein–ligand interactions.

Receptor	Ligand	RSMF	RSMD	rGyr	MolSA	SASA	PSA
4Q3T	5318767	2.017 ± 0.70	1.189 ± 0.38	4.270 ± 0.10	430.446 ± 11.42	295.193 ± 38.10	406.801 ± 11.76
	5318517	1.317 ± 0.55	1.151 ± 0.42	4.022 ± 0.13	316.260 ± 2.22	300.554 ± 63.60	178.618 ± 4.80
6UY4	5318517	0.895 ± 0.64	1.301 ± 0.36	3.728 ± 0.08	310.596 ± 240	99.332 ± 38.14	169.829 ± 4.90
	5280804	0.738 ± 0.44	0.580 ± 0.09	4.180 ± 0.03	356.443 ± 3.10	26.187 ± 9.24	383.859 ± 9.98
2X8H	5318767	1.365 ± 0.73	4.348 ± 0.59	4.265 ± 0.18	428.864 ± 10.88	434.628 ± 42.04	414.392 ± 10.01
	5318517	1.417 ± 0.84	1.137 ± 0.45	3.399 ± 0.13	315.950 ± 3.01	437.568 ± 134.21	179.797 ± 5.18

Note: values are represented as mean ± SEM measured in Armstrong units (Å). RMSD: root mean square deviation; RMSF: root mean square fluctuation; rGyr: radius of gyration; MolSA: molecular surface area; SASA: solvent accessibility surface area; PSA: pressure swing adsorption.

## Data Availability

The data presented in this study are available upon request from the corresponding author.
